# Contribution of Audiogram Classification in Evaluating Vestibular Dysfunction in Sudden Sensorineural Hearing Loss With Vertigo

**DOI:** 10.3389/fneur.2021.667804

**Published:** 2021-04-29

**Authors:** Zhuang Jiang, Jiajia Zhang, Ying Wang, Xuan Huang, Qingxiu Yao, Yanmei Feng, Shujian Huang, Hui Wang, Shankai Yin

**Affiliations:** ^1^Department of Otolaryngology-Head and Neck Surgery, Shanghai Jiao Tong University Affiliated Sixth People's Hospital, Shanghai, China; ^2^Otolaryngology Institute of Shanghai Jiao Tong University, Shanghai, China; ^3^Shanghai Key Laboratory of Sleep Disordered Breathing, Shanghai, China

**Keywords:** vertigo, sudden sensorineural hearing loss, audiogram configuration, vestibular end organ, vestibular dysfunction

## Abstract

**Object:** We aimed to identify the relationship between vertigo symptoms and the involvement of vestibular dysfunction in sudden sensorineural hearing loss (SSNHL) and the contribution of audiogram classification.

**Methods:** A total of 50 patients with unilateral SSNHL were retrospectively divided into the vertigo group and non-vertigo group depending on the presence of vertigo. The involved vestibular end organs (VEOs) were verified by a battery of vestibular function tests including video head impulse test (vHIT), cervical vestibular-evoked myogenic potential (cVEMP), and ocular VEMP (oVEMP). The correlations of audiogram configurations, initial pure-tone average (PTA), number of involved VEOs, prognosis (complete recovery rate), and vestibular functions were analyzed between the two groups. Additionally, the vestibular functions in a subgroup of profound SSNHL patients were further compared within groups with or without vertigo.

**Results:** Significant differences in the initial audiogram configurations (*p* = 0.033) and the abnormal rates of the posterior semicircular canal (PSC) (*p* = 0.035) and oVEMP (*p* = 0.046) were found between the two groups. The number of involved VEOs was related to the initial PTA in the vertigo group (*p* = 0.002, *r* = 0.541) and non-vertigo group (*p* = 0.042, *r* = 0.446). The prognosis was related to the abnormal rate of cVEMP and the number of involved VEOs in both vertigo group (*p* = 0.008, *r* = 0.482; *p* = 0.039, *r* = 0.385, respectively) and non-vertigo group (*p* = 0.016, *r* = 0.520; *p* = 0.022, *r* = 0.495, respectively), and it was especially related to the audiogram configurations in the vertigo group (*p* < 0.001, *r* = 0.692). However, after classification by audiogram configurations, there was no statistical difference in the abnormal rates of all vestibular function tests or the number of involved VEOs between the profound SSNHL patients with or without vertigo.

**Conclusion:** The relationship between the involvement of vestibular dysfunction and vertigo symptoms in patients with SSNHL was significantly different before and after audiogram classification. When evaluating the vestibular dysfunction in SSNHL patients, more attention should be paid to the audiogram configuration.

## Introduction

Sudden sensorineural hearing loss (SSNHL) is a serious otological disorder with an annual incidence of 2.4–27 people per 100,000 people (1, 2). It is defined as a 30-dB sensorineural hearing loss occurring in three contiguous frequencies, or at least loss in two adjacent frequencies of ≥20 dB and developing over 3 days (3). The configuration of hearing loss can affect high, low, or all frequencies, and audiograms are accordingly classified into low-frequency, high-frequency, flat-type, and profound hearing loss (3). The pathology of SSNHL is still unknown (4), and the prognosis of SSNHL patients can be determined by the patient's age, the time between hearing loss onset and treatment, vertigo accompanying the hearing loss, and type of hearing loss, among other factors (5, 6). Approximately 30–40% of SSNHL patients suffer from vestibular symptoms besides cochlear dysfunction, while additional symptoms of tinnitus and fullness are reported in 80% of cases (7, 8). Vestibular involvement is more common in patients with profound hearing loss, in the form of dizziness, unsteadiness, or even rotatory vertigo (9). However, the pathophysiology of this condition has not been identified, but several reports have been proposed.

Vestibular dysfunction in SSNHL is considered an extension of the disease caused by the proximal anatomical correlation of the vestibule and cochlea, which contribute to more severe inner ear damage (9). In recent years, the assessment of peripheral vertigo includes many tests, such as the rotatory caloric test, video head impulse test (vHIT), cervical vestibular-evoked myogenic potential (cVEMP), and ocular VEMP (oVEMP) (10–12). Some studies have demonstrated the characteristic and vestibular test outcomes in SSNHL patients with vertigo. Iwasaki et al. suggested that the saccule was involved more frequently than the horizontal semicircular canal (HSC) in patients with idiopathic sudden hearing loss with vertigo (13). Lim et al. demonstrated that the functions of vestibular organs, particularly the utricle and HSC, are associated with disease severity and hearing outcome in patients with SSNHL (14). Byun et al. studied the function of each semicircular canal and saccule using vHIT, cVEMP, and caloric tests, and found that abnormal vHIT gain in the posterior semicircular canal (PSC) appears to be a specific prognostic factor for incomplete hearing recovery in SSNHL (15). Various inconsistent results were presented regarding involved vestibular dysfunction in patients with SSNHL depending on the presence of vertigo.

Although a number of studies have described the involvement of vestibular dysfunction in SSNHL patients with vertigo, results have varied widely (16–19). One reason for this dissimilarity is that related studies did not consider hearing classification, and the included groups were heterogeneous. It is well-known that SSNHL patients with different types of audiogram configurations have obvious differences in the hearing recovery (5, 9, 20). Furthermore, different types of audiogram configurations may indicate different pathogeneses according to German SSNHL guidelines (21). Vestibular dysfunction involvement presenting with vertigo is more common in patients with profound hearing loss (19, 20, 22). Thus, studies on vestibular function tests in SSNHL with or without vertigo are indispensable according to their initial pure-tone average (PTA) and it is necessary to study the vestibular function in profound SSNHL patients.

This study aimed to explore the relationship between vertigo symptoms and the involvement of vestibular dysfunction in SSNHL patients and the contribution of audiogram classification based on the results of a battery of vestibular function tests targeting different vestibular end organs.

## Materials and Methods

### Participants

This study retrospectively reviewed the clinical data of 50 patients (26 women, 24 men; 53.80 ± 14 years) with unilateral SSNHL who visited the ear–nose–throat clinic of the Sixth People's Hospital affiliated to the Shanghai Jiao Tong University from November 2018 to June 2020. The SSNHL diagnosis and treatment guidelines were established by the Chinese Medical Association of Otorhinolaryngology, Head and Neck Surgery in 2015 (3). A comprehensive medical history was taken to obtain demographic and clinical information, including associated symptoms such as tinnitus, vertigo, or aural fullness, the duration of vertigo or dizziness, and provocation factors. An otoscopic examination was performed to rule out abnormalities of the external or middle ear. The inclusion criteria included (1) age ≥ 18 years; (2) unilateral SSNHL according to the 2015 guideline; (3) an unknown cause; (4) no fluctuation in hearing loss; and (5) initiation of treatment within 14 days after onset. The exclusion criteria were as follows: hearing loss due to other definite causes such as otitis media, Meniere's disease, otosclerosis, congenital deafness, presbycusis, vestibular schwannoma, and inner ear malformation. Patients with insufficient medical records and examination data were also excluded. Patients were divided into two groups: vertigo group (*n* = 29) and non-vertigo group (*n* = 21). After recruitment, patients were asked to complete an informed consent form and underwent audiometry and vestibular function tests, which were evaluated on two scheduled mornings with a battery including vHIT and VEMP tests within 1 week after the first visit.

### Audiometry

Pure-tone audiometry was performed by qualified medical assistants in a soundproof room. Audiograms were measured with a manual audiometer (GSI-61; Grason-Stadler, Eden Prairie, MN) coupled with TDH-39 headphones in one-octave steps at frequencies ranging from 0.25 to 8 kHz. According to the 2015 guidelines for diagnosis and treatment of sudden deafness, audiographic results were categorized as low-frequency, high-frequency, flat-type, or profound hearing loss in terms of the frequency and degree of hearing loss. The PTA was calculated as the average of the thresholds of the impaired frequency.

### vHIT

Three-dimensional vHIT was performed using ICS Impulse® 3.0 (Natus Medical Denmark ApS). All tests were carried out by an experienced inspector according to standard protocols proposed by Halmagyi (23). During the test, subjects were seated 1 m directly in front of a fixation target at eye level, and the operator held the subject's head from behind and performed a short and rapid operation in both horizontal and vertical positions. Passive impact exercises were performed more than 20 times (amplitude 5–10°, peak angular velocity 200–250°/s), the time and direction of which should not be predicted by the subject. The angular velocity of the eye movement head was recorded, and the ratio of the compensated slow-phase eye velocity to the head pulse velocity is calculated as the vestibulo-ocular reflex gain (24). The normal values of the gain of the HSC are 0.8–1.2, and the gain of the anterior semicircular canal (ASC) and PSC are 0.7–1.2 (23).

### VEMP

A two-channel VEMP system (Neuro-Audio; Neurosoft, Ivanovo, Russia) was used to evaluate VEMPs in a comfortable soundproof room. VEMPs are short-latency potentials evoked by air-conducted sound. cVEMPs are easily recorded in the cervical muscles and can also evaluate saccular and vestibulo-ocular reflexes (25, 26). The recording electrode was located at the midpoint of the sternocleidomastoid (SCM) muscle on both sides, the reference electrode was positioned on the upper end of the sternum, and the ground electrode was placed in the middle of the forehead. Subjects were asked to raise their head to keep the SCM muscle in a rigid contraction state. cVEMPs are widely employed for the functional assessment of the saccular and inferior vestibular nerves. The recording electrode was located ~1 cm from the midpoint of the inferior rim of the bilateral orbits, the reference electrode was placed 2–3 cm below it, and the ground electrode was positioned between the eyebrows. Skin surface impedance was <5 kΩ.

Using a tone-burst stimulus of 500 Hz and 100 dB nHL through inserted earphones (IP30), the stimulation frequency was five times per second (the recording time window was 100 ms), and the bandpass filter range was 30–2000 Hz. After 5000× magnification processing, the scanning number was 64~256, and the potential change generated was recorded. In general, a typical VEMP waveform includes an initial positive peak (P1) and a subsequent negative peak (N1). The oVEMP and cVEMP recorded by the SCM muscle and external ocular surface electrodes were calculated. The peak amplitude of the oVEMP waveform was calculated between P1 and N1. The Interaural Asymmetry Ratio (IAR) was calculated as follows: (amplitude of the unaffected ear – amplitude of the affected ear)/(amplitude of the unaffected ear + amplitude of the affected ear) × 100%. An absolute value of IAR >29% or the absence of a response on the affected side suggested saccule and/or inferior vestibular nerve involvement. cVEMP can be used to investigate the function of the saccule and the inferior vestibular nerve, and oVEMP can be used to assess the function of the utricle and the superior vestibular nerve (27).

### Evaluation of Vestibular End Organ Involvement

The inner ear is divided into five organs including the saccule, utricle, HSC, ASC, and PSC. The involvement of each vestibular organ was verified by abnormalities in any vestibular test(s) targeting this organ.

### Treatments and Hearing Outcomes

All patients had been hospitalized, and all had received systemic steroid therapy with intravenous methylprednisolone at a decreasing dose of 80 mg for 5 days followed by 40 mg for 5 days aided with vasodilators and neurotrophic drugs. After discharge, patients were prescribed oral vasodilators and neurotrophic drugs for 1 month. The follow-up period was 1 month. Hearing recovery was graded as complete recovery (a return of hearing to normal range or the level of the unaffected contralateral ear), partial recovery (improvement by more than 30 dB), slight recovery (improvement by more than 15 dB but not within 30 dB), or no response (either an improvement of 15 dB or less, no change, or deterioration) according to the guideline (3). Further, a prognosis was judged based on the complete recovery rate (3).

### Statistical Analysis

Data were analyzed using SPSS Statistics (version 24.0 for Windows; IBM, Armonk, NY). Descriptive data are presented as means and standard deviations (SD) or median and interquartile range (IQR). Categorical data are expressed as number and percentage and were compared using Pearson's χ^2^ or Fisher exact test. The *t*-test or Mann–Whitney U test was used to compare continuous variables, if appropriate. Spearman rank or Pearson correlation analysis was used for the correlation analysis of two variables. A difference of *p* < 0.05 was regarded as significant.

## Results

**Figure 1:**
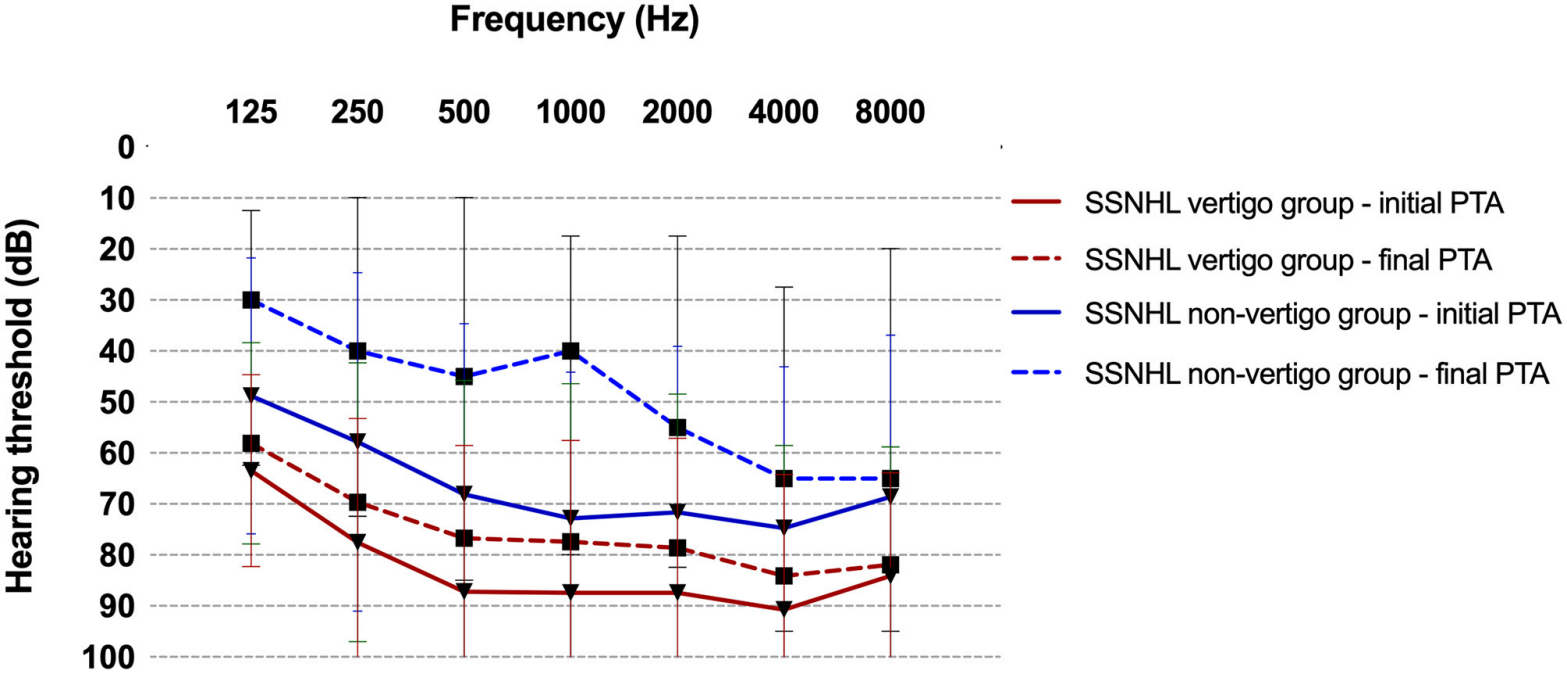
The difference between the initial and final PTA in the SSNHL vertigo group and non-vertigo group. There was no significant difference in hearing recovery rate between the two groups (*p* = 0.558). SSNHL, sudden sensorineural hearing loss; PTA, pure-tone average.

Among the 50 patients, the youngest was 18 years old and the oldest was 75 years old (53.80 ± 14 years). The average ages of subjects in the vertigo group (*n* = 29) and non-vertigo group (*n* = 21) were 55.93 ± 14.15 and 50.86 ± 15.81 years, respectively. There was no difference in age (*p* = 0.24), gender distribution (*p* = 0.077), or affected ear (*p* = 0.802) between subjects with SSNHL with or without vertigo. The details of clinical data and audio-vestibular tests are described in Table 1. The mean initial PTA and final PTA were found to be worse in subjects with vertigo (84.58 ± 20.22 dB, 76.41 ± 22.94 dB; respectively) than in subjects without vertigo (72.01 ± 20.55 dB, 52.24 ± 30.93 dB; respectively) (*p* = 0.007, *p* = 0.006; respectively). There was a significant difference between the initial audiogram configurations (*p* = 0.033), indicating that more serious hearing loss was seen in the vertigo group (86.2% presented with profound hearing loss) than the non-vertigo group (47.6% presented with profound hearing loss). However, there was no difference in hearing recovery between the two groups (*p* = 0.569) (Figure 1).

**Table 1:** 

	**Vertigo group (*n* = 29)**	**Non-vertigo group (*n* = 21)**	***P*-value**
Age (years)	55.93 ± 14.15	50.86 ± 15.81	0.240
Sex			0.077
Male % (*n*)	41.4 (12)	66.7 (14)	
Female % (*n*)	58.6 (17)	33.3 (7)	
Affected side			0.774
Center % (*n*)	48.3 (14)	52.4 (11)	
Right % (*n*)	51.7 (15)	47.6 (10)	
Abnormal HSC % (*n*)	6.9 (2)	14.3 (3)	0.638
Abnormal ASC % (*n*)	34.5 (10)	33.3 (7)	0.933
Abnormal PSC % (*n*)	58.6 (17)	28.6 (6)	0.035^*^
Abnormal cVEMP % (*n*)	75.9 (22)	61.9 (13)	0.288
Abnormal oVEMP % (*n*)	82.8 (24)	57.1 (12)	0.046^*^
Number of involved vestibular end organs	2.55 ± 1.40	1.76 ± 1.34	0.093
Initial PTA (dB)	84.58 ± 20.22	72.01 ± 20.55	0.007^**^
Final PTA (dB)	76.41 ± 22.94	52.24 ± 30.93	0.006^**^
Audiogram configuration % (*n*)			0.019^*^
Low-frequency hearing loss	3.4 (1)	14.3 (3)	
High-frequency hearing loss	3.4 (1)	9.5 (2)	
Flat-type hearing loss	6.9 (2)	28.6 (6)	
Profound hearing loss	86.2 (25)	47.6 (10)	
Hearing recovery % (*n*)			0.558
Complete recovery	6.9 (2)	14.3 (3)	
Partial recovery	13.8 (4)	23.8 (5)	
Slight recovery	13.8 (4)	14.3 (3)	
No response	65.5 (19)	47.6 (10)	

Continuous data are presented as mean ± SD values; categorical data are reported as percentage (number). The asterisk indicates statistical significance between groups (*p < 0.05, **p < 0.01).

*HSC, horizontal semicircular canal; ASC, anterior semicircular canal; PSC, posterior semicircular canal; cVEMP, cervical vestibular-evoked myogenic potential; oVEMP, ocular vestibular-evoked myogenic potential; PTA, pure-tone average*.

**Figure 2:**
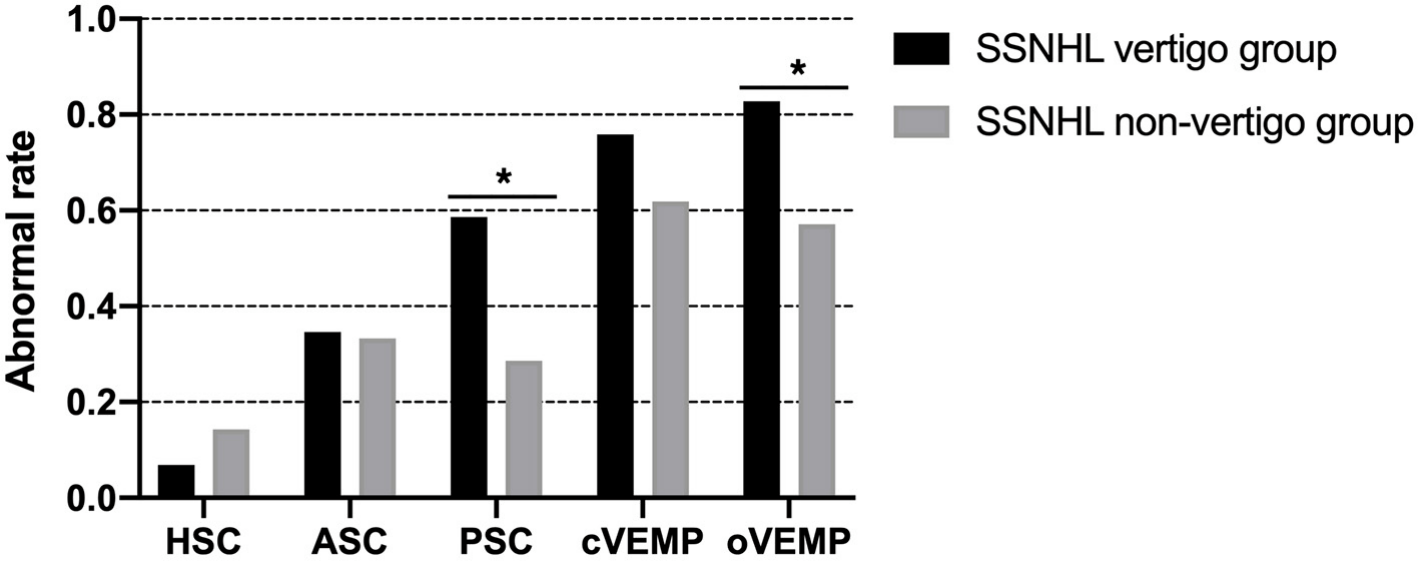
The abnormal rates of vestibular tests of the SSNHL vertigo group and non-vertigo group. Significant differences in the abnormal rate of the PSC (*p* = 0.035) and oVEMP (*p* = 0.046) were found between groups. *Indicates significant (*p* < 0.05) difference in vestibular function test between groups. SSNHL, sudden sensorineural hearing loss; HSC, horizontal semicircular canal; ASC, anterior semicircular canal; PSC, posterior semicircular canal; cVEMP, cervical vestibular-evoked myogenic potential; oVEMP, ocular vestibular-evoked myogenic potential.

The vertigo group showed a higher abnormal rate of oVEMP (82.8%) and cVEMP (75.9%), followed by vHIT for the PSC (58.6%), ASC (34.5%), and HSC (6.9%). In the non-vertigo group, the abnormal rate was highest in cVEMP (61.9%), followed by oVEMP tests (57.1%) and vHIT for the ASC (33.3%), PSC (28.6%), and HSC (14.3%). Significant differences in abnormalities in the PSC (*p* = 0.035) and oVEMP (*p* = 0.046) were found between the two groups. The abnormal rates of vestibular tests of both groups are shown in Figure 2. The median number of involved VEOs in the vertigo group was 3 [interquartile range (IQR), 2–4] with an average of 2.59. The median number of involved VEOs in the non-vertigo group was 2 (IQR, 1–3) with an average of 1.95. There was no significant difference in the number of involved VEOs between the two groups (*p* = 0.093).

**Figure 3:**
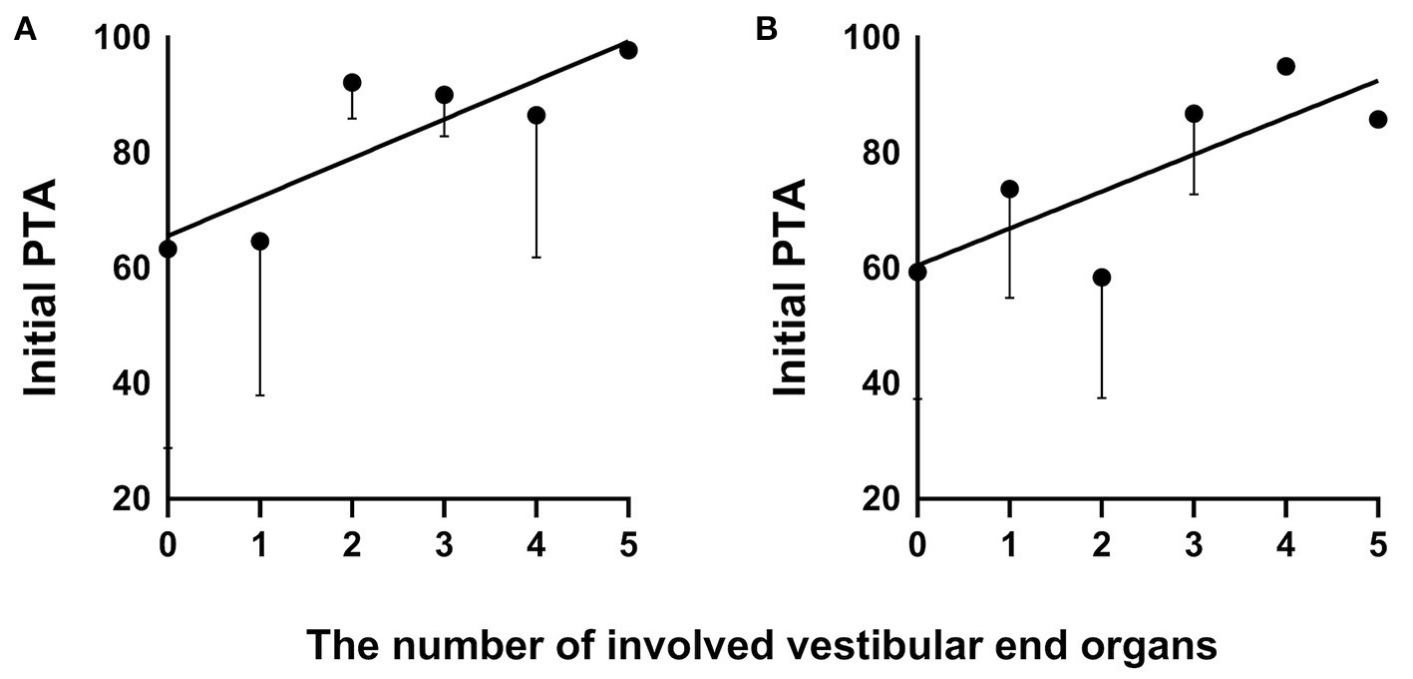
The correlation of the number of involved vestibular end organs and initial PTA in the SSNHL vertigo group **(A)** and non-vertigo group **(B)**. SSNHL, sudden sensorineural hearing loss; PTA, pure-tone average.

**Figure 4:**
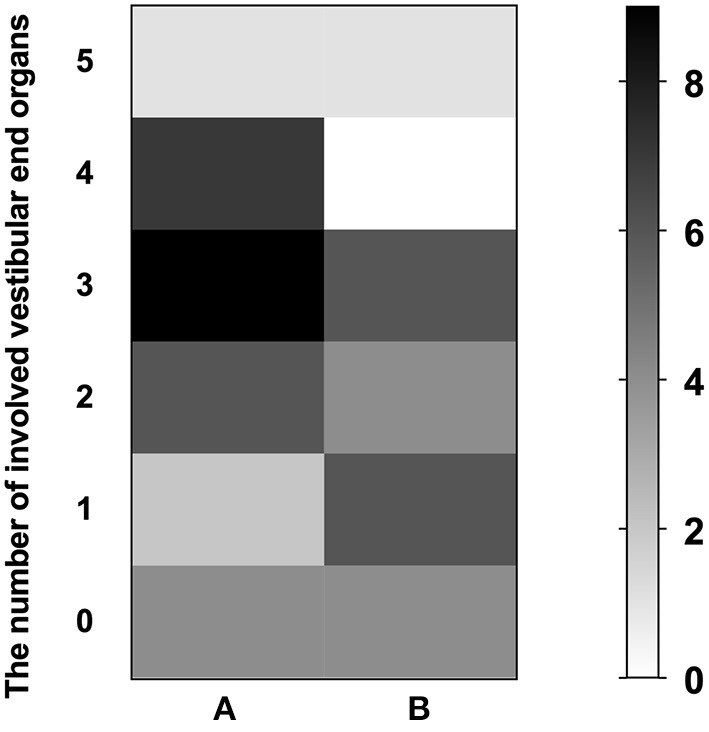
The numbers of patients with involved vestibular end organs (VEOs) in the SSNHL vertigo group **(A)** and non-vertigo group **(B)**. The left ordinate represents the number of involved VEOs; the vertical axis on the right represents the number of patients.

In the vertigo group, the prognosis for the hearing outcome was related to the abnormal rate of cVEMP (*p* = 0.008, *r* = 0.482, Spearman rank), the number of involved VEOs (*p* = 0.039, *r* = 0.385, Spearman rank), initial PTA (*p* = 0.027, *r* = 0.411, Spearman rank), and audiogram configurations (*p* < 0.001, *r* = 0.692, Spearman rank). In the non-vertigo group, the prognosis for the hearing outcome was related to the abnormal rate of cVEMP (*p* = 0.016, *r* = 0.520, Spearman rank) and the number of involved VEOs (*p* = 0.022, *r* = 0.495, Spearman rank). In addition, we analyzed the relationship between the number of involved VEOs and initial PTA in the vertigo group (*p* = 0.002, *r* = 0.541, Pearson) (Figure 3A) and the non-vertigo group (*p* = 0.042, *r* = 0.446, Pearson) (Figure 3B). This suggests that profound hearing loss shown by initial PTA always involves more VEOs. The numbers of patients with involved VEOs and in the two groups are shown in Figure 4.

**Figure 5:**
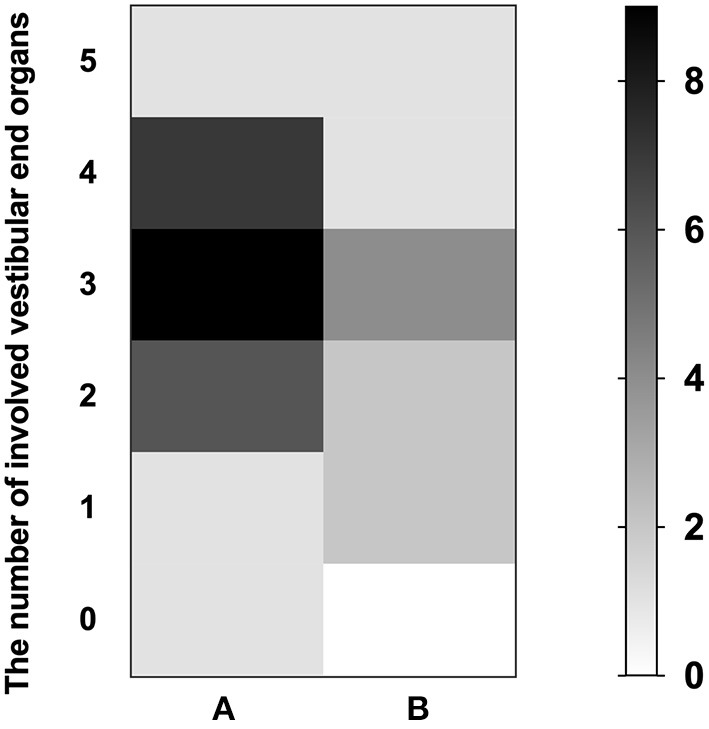
The number of patients with involved vestibular end organs (VEOs) in the subgroup of profound SSNHL patients with vertigo group **(A)** and without vertigo **(B)**. The left ordinate represents the number of involved VEOs; the vertical axis on the right represents the number of patients.

In addition, we identified the contribution of VEOs and audiogram configuration to the prognosis in a subgroup of patients with profound SSNHL. The details of the audio-vestibular tests in patients with profound SSNHL are described in Table 2. Higher abnormal rates of cVEMP (90%) and oVEMP (90%) were found in subjects with profound SSNHL without vertigo, but there was no significant difference in abnormal rates of cVEMP and oVEMP between the two subgroups (*p* = 0.867, *p* = 0.867) or in the abnormal rates of HSC (*p* = 0.561), ASC (*p* = 0.709), or PSC (*p* = 0.151). The median number of involved VEOs in subjects with profound SSNHL with vertigo was 3 (IQR, 2–4) with an average of 2.92, the median number of involved VEOs in subjects with profound SSNHL but no vertigo was 3 (IQR, 1.75–3.25) with an average of 2.6, and no significant difference was found between the two groups (*p* = 0.553). The mean initial PTA was similar in those with profound SSNHL and vertigo (90.47 ± 13.73) than in those with profound SSNHL without vertigo (82.27 ± 18.46) (*p* = 0.090). There was no difference in hearing recovery between the two subgroups (*p* = 0.220), but 68.0% of subjects with vertigo did not respond to treatment. The number of patients with involved VEOs in the two subgroups is shown in Figure 5. Furthermore, no significant correlation was observed between initial PTA and the number of involved VEOs in the two subgroups.

**Table 2:** The audio-vestibular tests in the patients with profound sudden sensorineural hearing loss.

	**With vertigo (*n* = 25)**	**Without vertigo (*n* = 10)**	***P*-value**
Abnormal HSC % (*n*)	8.0 (2)	20 (2)	0.561
Abnormal ASC % (*n*)	40.0 (10)	30.0 (3)	0.709
Abnormal PSC % (*n*)	68.0 (17)	40 (4)	0.151
Abnormal cVEMP % (*n*)	88.0 (22)	90.0 (9)	0.867
Abnormal oVEMP % (*n*)	88.0 (22)	90.0 (9)	0.867
Number of involved vestibular end organs	2.92 (±1.12)	2.60 (±1.17)	0.553
Initial PTA (dB)	90.47 (±13.73)	82.27 (±18.46)	0.090
Hearing recovery % (*n*)			0.220
Complete recovery	0 (0)	0 (0)	
Partial recovery	16.0 (4)	40.0 (4)	
Slight recovery	16.0 (4)	20.0 (2)	
No response	68.0 (17)	40.0 (4)	

*Continuous data are presented as mean ± SD values; categorical data are reported as percentage (number)*.

*HSC, horizontal semicircular canal; ASC, anterior semicircular canal; PSC, posterior semicircular canal; PTA, pure-tone average; cVEMP, cervical vestibular-evoked myogenic potential; oVEMP, ocular vestibular-evoked myogenic potential*.

## Discussion

In the present study, we compared the hearing and vestibular dysfunction outcome between SSNHL patients with or without vertigo. Significant differences in initial audiogram configurations and the abnormal rates of PSC and oVEMP were found between two groups, indicating that more serious hearing loss was seen in the vertigo group than non-vertigo. The number of involved VEOs was related to the initial PTA in the two groups. Despite statistical differences in initial PTA between the two groups, the number of involved VEOs did not differ between the two groups. There was no difference in hearing recovery between the two groups. The prognosis of SSNHL was related to the abnormal rate of cVEMP and the number of involved VEOs in both vertigo group and non-vertigo group, and it was especially related to the audiogram configurations in the vertigo group. However, when we classified SSNHL patients by audiogram configurations, there was no significant difference in the abnormal rates of the HSC, ASC, or PSC or in the involved number of VEOs between profound SSNHL patients with and without vertigo.

It is generally believed that the cochlea and vestibule share a continuous membrane structure and similar receptor cell superstructure and may be susceptible to the same harmful factors. Inner ear diseases such as sudden hearing loss can cause vertigo (28). Previous studies have shown that cochlear impairment could be more serious in SSNHL patients with vertigo than in those without, and our results are consistent with those studies (14, 29, 30), and more serious hearing loss was seen in SSNHL patients with vertigo. Anatomically, the base cochlear turns are closer to the terminal vestibular organs than the frontal cochlear turns (7, 13), and the anterior vestibular artery provides blood supply to the LSCC, ASCC, utricle, and saccule. Studies reported that patients with high-frequency hearing loss tend to suffer more vertigo attacks (31), which could be diagnosed as “vestibulo-cochlear artery syndrome” (32), although a study suggested that audio-vestibular tests did not provide easy separation between ischemic and non-ischemic causes of vertigo with SSNHL (12). In the case of partial frequency or mild to moderate hearing loss, the patient can make up for the results caused by overall adjustment or compensation, so as not having vertigo. However, some research showed that patients with low-frequency hearing loss may also have more vertigo attacks, which may be because low-frequency hearing loss is related to endolymphatic hydrops (16, 17, 33). In our results, patients with profound SSNHL tended to suffer more vertigo attacks, which may be due to the small number of cases of low- and high-frequency sudden hearing loss in our study. This suggests that the anatomical position of the cochlea may not fully explain the relationship between vestibular organ involvement and vestibular dysfunction and cochlear damage in patients with SSNHL.

In our study, we found that VEOs may be impaired not only in SSNHL patients with vertigo but also in those without vertigo. The results were consistent with previously reported results (19, 31). It is generally accepted that cochlear and vestibular impairment could be more serious in patients with vertigo than in those without vertigo (16). However, in this study, it is worth noting that there was no significant difference in the number of involved VEOs between the two groups (*p* = 0.093). In other words, the number of involved VEOs in SSNHL patients with vertigo was similar to that in SSNHL patients without vertigo. Liu et al. reported that the abnormal rate of the oVEMP test was the highest in SSNHL patients, followed by abnormal rates of cVEMP tests, regardless of the presence of vertigo, indicating that the utricle might be more prone to damage than the saccule (16). Our results were consistent with the findings of Liu et al. in the vertigo group, but the results of the non-vertigo group were opposite, where the cVEMP test had the highest abnormal rate followed by abnormal rates of oVEMP tests, and the abnormal rate of oVEMP tests was significantly different between the two groups. Furthermore, a significant difference in the abnormal rate of the PSC was found between SSNHL patients with or without vertigo in our study. Studies have also suggested that vestibular dysfunction is likely to extend from organs close to the cochlea to organs farther from the cochlea (15, 27). In our opinion, the reason for the inconsistent results may be the different audiogram configurations used for SSNHL patients.

In addition, a correlation (*p* < 0.01) was found between the initial PTA and the number of involved VEOs, not only in SSNHL patients with vertigo but also in those without vertigo. Consistent with our results in patients without vertigo, Lim et al. found that utricular dysfunction is associated with the severity of initial hearing (14). In our study, although there were statistical differences in the initial PTA of the two groups, there was no difference in the number of involved VEOs. It is clear that hearing loss affects the function of VEOs, but involved VEOs does not necessarily cause vertigo symptoms.

Vertigo is a well-recognized prognostic indicator, as it is correlated with poor hearing recovery. Our results show that SSNHL patients with vertigo have a worse prognosis, while there was no difference in whole rate of hearing recovery between the two groups. However, the prognosis was closely related to audiogram configurations just in the vertigo group, which may due to a high proportion of profound SSNHL patients (86.2%); this hints that more attention should be paid to audiogram configuration when evaluating the recovery of hearing outcomes except the vertigo symptoms. In the present study, we also attempted to assess the impact of various variables on the prognosis of hearing outcomes. In agreement with previous studies, vestibular abnormalities are associated with more severe hearing loss and indicate a poorer prognosis (14, 31, 34–36).

Furthermore, to clear the necessity of classifying SSNHL by audiogram configuration, we separately analyzed the vestibular function of patients with profound SSNHL. As discussed before (14, 29, 31), cochlear impairment, initial PTA, and hearing recovery were much worse in patients with vertigo. In those with profound SSNHL with or without vertigo, higher abnormal rates of oVEMP (88–90%) and cVEMP (88–90%) were found but with no significant differences between the two subgroups, respectively. This suggests that profound SSNHL patients may have suffered saccule and utricle damage no matter whether with or without vertigo; in other words, the value of these vestibular tests to the profound SSNHL is limited. One earlier report investigated the histopathologic characteristics of the temporal bone in SSNHL with and without vertigo and found no direct relationship between the presence of vertigo and damage to the vestibular apparatus. Vertigo associated with sudden deafness may be due to the transmission of biochemical changes in the inner ear fluid from the cochlea to the vestibular apparatus (37, 38). These findings suggest that vertigo may not necessarily bear a causal relationship with the impairment of the VEOs, especially in patients with profound SSNHL, reemphasizing the importance of the classification of SSNHL by audiogram in the evaluation of vestibular function in patients with SSNHL.

The major limitation of this study is that the sample size is small, and each type of PTA varies greatly, which may limit accurate conclusions. In addition, our study only discusses the relationship between vertigo and vestibular function impairment in patients with profound SSNHL and does not analyze the other three types after audiogram classification. Further studies will include more types of cases to determine the importance of classifying SSNHL by audiogram configuration to evaluate the involvement of vestibular organs in SSNHL patients with vertigo.

## Conclusions

Our study demonstrates that the vestibular dysfunction of SSNHL patients with or without vertigo is heterogeneous. The number of involved VEOs was related to initial PTA. However, there was no significant difference in the abnormal rates of vestibular function or the involved number of VEOs in the profound SSNHL patients no matter whether they are accompanied by vertigo, which hints that when evaluating involved vestibular dysfunction and prognosis in the SSNHL patients, more attention should be paid to the audiogram configuration.

## Data Availability Statement

The raw data supporting the conclusions of this article will be made available by the authors, without undue reservation.

## Ethics Statement

The studies involving human participants were reviewed and approved by Institutional Ethics Review Board of the Shanghai Sixth People's Hospital. The patients/participants provided their written informed consent to participate in this study.

## Author Contributions

Study conception and design: HW and SH. Acquisition of data: JZ, YW, XH, QY, and YF. Analysis and interpretation of data and drafting of manuscript: ZJ and JZ. Critical revision: HW and SY. All authors read and approved the final manuscript.

## Conflict of Interest

The authors declare that the research was conducted in the absence of any commercial or financial relationships that could be construed as a potential conflict of interest.

## Abbreviations

SSNHL: sudden sensorineural hearing loss
VEOs: vestibular end organs
vHIT: video head impulse test
cVEMP: cervical vestibular-evoked myogenic potential
oVEMP: ocular VEMP
PSC: posterior semicircular canal
HSC: horizontal semicircular canal
ASC: anterior semicircular canal
IAR: interaural asymmetry ratio
PTA: pure-tone average.
